# BoALMT1, an Al-Induced Malate Transporter in Cabbage, Enhances Aluminum Tolerance in *Arabidopsis thaliana*

**DOI:** 10.3389/fpls.2017.02156

**Published:** 2018-01-23

**Authors:** Lei Zhang, Xin-Xin Wu, Jinfang Wang, Chuandong Qi, Xiaoyun Wang, Gongle Wang, Mingyue Li, Xingsheng Li, Yang-Dong Guo

**Affiliations:** ^1^Beijing Key Laboratory of Growth and Developmental Regulation for Protected Vegetable Crops, College of Horticulture, China Agricultural University, Beijing, China; ^2^Shandong Huasheng Agriculture Co. Ltd, Shandong, China

**Keywords:** aluminum tolerance, *BoALMT1*, cabbage, malates, SIET

## Abstract

Aluminum (Al) is present in approximately 50% of the arable land worldwide and is regarded as the main limiting factor of crop yield on acidic soil. Al-induced root malate efflux plays an important role in the Al tolerance of plants. Here, the aluminum induced malate transporter *BoALMT1* (KF322104) was cloned from cabbage (*Brassica oleracea*). *BoALMT1* showed higher expression in roots than in shoots. The expression of *BoALMT1* was specifically induced by Al treatment, but not the trivalent cations lanthanum (La), cadmium (Cd), zinc (Zn), or copper (Cu). Subcellular localization studies were performed in onion epidermal cells and revealed that BoALMT1 was localized at the plasma membrane. Scanning Ion-selective Electrode Technique was used to analyze H^+^ flux. *Xenopus* oocytes and *Arabidopsis thaliana* expressing *BoALMT1* excreted more H^+^ under Al treatment. Overexpressing *BoALMT1* in transgenic *Arabidopsis* resulted in enhanced Al tolerance and increased malate secretion. The results suggested that *BoALMT1* functions as an Al-resistant gene and encodes a malate transporter. Expressing *BoALMT1* in *Xenopus* oocytes or *A. thaliana* indicated that BoALMT1 could increase malate secretion and H+ efflux to resist Al tolerance.

## Introduction

Al is the most abundant metal and the third most abundant element, making up around 7% of the earth's crust (Tesfaye et al., 2001). When the soil pH value is lower than 5.0, the soluble aluminum in soil solutions is mostly present as the toxic Al^3+^, which inhibits root growth at micromolar concentrations in many species (Kochian et al., 2005). Micromole levels of Al^3+^ can remarkably inhibit root elongation, and impair the absorption, of water and nutrients (Kochian et al., 2005). The well-known mechanism of plant Al tolerance is the Al-induced secretion of organic acids (OA) from the root tips. The OAs chelate Al^3+^ and form the non-toxic compound OA-Al (Kochian et al., 2004; Horst et al., 2010; Ryan et al., 2011). The most common OAs involved in the Al detoxification process are malate, citrate, and oxalate, depending on the plant. For example, malate is used in wheat (Delhaize et al., 1993) and *Arabidopsis* (Hoekenga et al., 2003), citrate is secreted in maize (Pellet et al., 1995), and oxalate is used in buckwheat (Zheng et al., 2005) and tomato (Yang et al., 2008).

Wheat *TaALMT1* (ALMT, for Al-activated Malate Transporter) encoding a malate transporter was the first plant gene involved in Al tolerance and the first ALMT family gene. In Al-tolerant wheat genotypes, *TaALMT1* is specifically expressed in the root tips (Sasaki et al., 2004; Raman et al., 2005). Overexpression of *TaALMT1* in wheat, barley, and tobacco-cell suspension increases the efflux of Al-activated malate and enhances tolerance to Al stress (Delhaize et al., 2004; Sasaki et al., 2004; Pereira et al., 2010). *TaALMT1* homologs have now been isolated in *Arabidopsis* (Hoekenga et al., 2006), oilseed rape (Ligaba et al., 2006), rye (Collins et al., 2008), soybean (Liang et al., 2013), and *Medicago sativa* (Chen et al., 2013). Multi-antimicrobial extrusion (MATE) proteins are a family of proteins that function as drug/sodium or proton antiporters. MATE proteins can secrete organic anions to contribute to the Al tolerance in plants (Furukawa et al., 2007; Magalhaes et al., 2007; Wang et al., 2007; Liu et al., 2009). In *Arabidopsis*, the zinc finger transcription factor *STOP1* (known as ART1 in rice) plays a critical role in plant Al tolerance by regulating the Al-inducible expression of *ALMT* and *MATE* (Liu et al., 2009). In rice, multiple genes implicated in Al tolerance, including MATE transporter family members, are regulated by the transcription factor ART1 (Yamaji et al., 2009).

Cabbage (*Brassica oleracea*) is one of the most important vegetable crops around the world (Wu et al., 2014). Our previous study has shown that *BoMATE* encodes a citrate transporter and is induced by Al and enhances aluminum tolerance in *Arabidopsis* (Wu et al., 2014). Here we report that cabbage BoALMT1 is located in the plasma membrane and induced by Al. A reverse genetic approach was used to characterize the functions of *BoALMT1*. Overexpressing *BoALMT1* in *Xenopus oocytes and Arabidopsis* facilitated H^+^ efflux. Overexpressing *BoALMT1* in *Arabidopsis* resulted in enhanced Al tolerance and increased malate secretion. These results suggested that BoALMT1 has an important role in Al tolerance in cabbage.

## Results

### Sequence analysis of *BoALMT1* in cabbage

**Figure 1:**
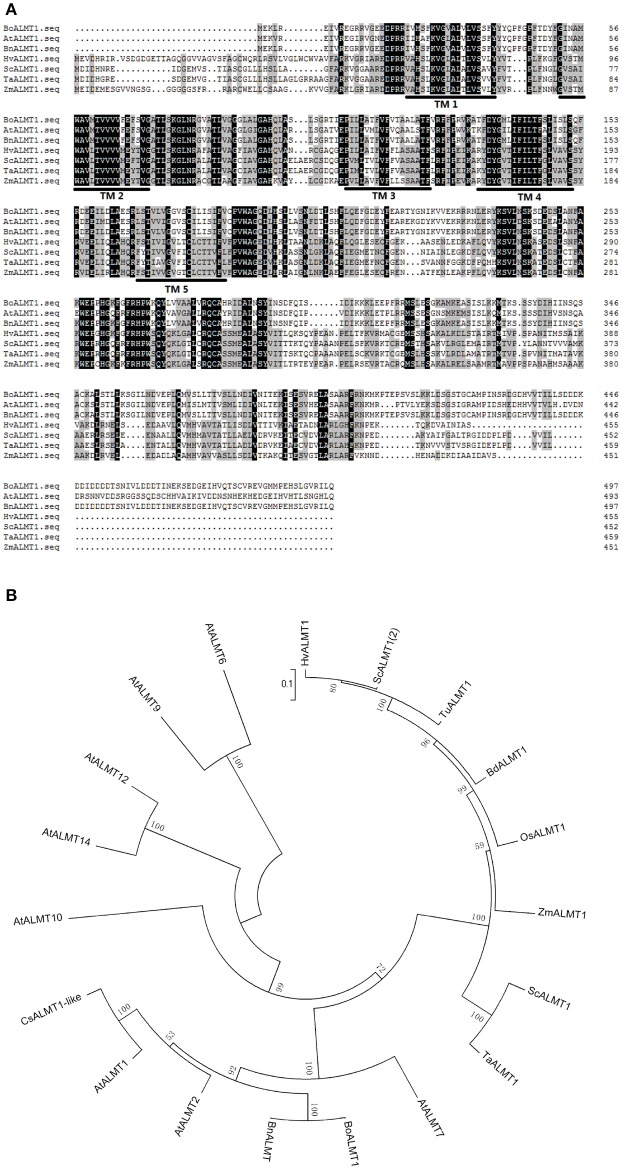
Amino acid sequence **(A)** and phylogenetic **(B)** analysis of *Brassica oleracea BoALMT1*. **(A)** Multiple sequence alignment of cabbage *BoALMT1*, maize *ZmALMT1*, wheat *TaALMT1, Arabidopsis AtALMT1*, rape *BnALMT1*, rye *ScALMT1*, and Barley *HvALMT1*. Identical amino acids and similar amino acids were indicated by dark shading and light shading, respectively. Lines depict the 5 predicted transmembrane domains in BoALMT1 as predicted by HMMTOP. **(B)** Phylogenetic relationship of BoALMT1 and other known Al-activated malate transporters (ALMT). The amino acid sequences were aligned by ClustalW.

The ALMT gene was the first Al^3+^ tolerance gene identified in plants (Sasaki et al., 2004; Delhaize et al., 2007; Meyer et al., 2010). Membrane protein ALMTs possess 5–7 predicted transmembrane domains and a UPF0005 domain with unknown function (Delhaize et al., 2007). *BoALMT1* (KF322104) cloned from cabbage contains an open reading frame of 1,497 bp, encoding a polypeptide of 498 amino acids. BLAST analysis revealed that the sequence of BoALMT1 was a 99% match to BnALMT1 from rape, 73% match to AtALMT1 from *Arabidopsis*, and 33% match to TaALMT1 from wheat. The HMMTOP transmembrane topology prediction server was used to predict the localization of helical transmembrane segments and the analysis indicated that BoALMT1 contained 5 predicted transmembrane domains (Figure 1A). Analysis of BoALMT1 and other reported ALMTs in plants indicated that BoALMT1 was most closely clustered with the BnALMT1 from *Brassica napus* (Figure 1B).

### Expression pattern of *BoALMT1*

**Figure 2:**
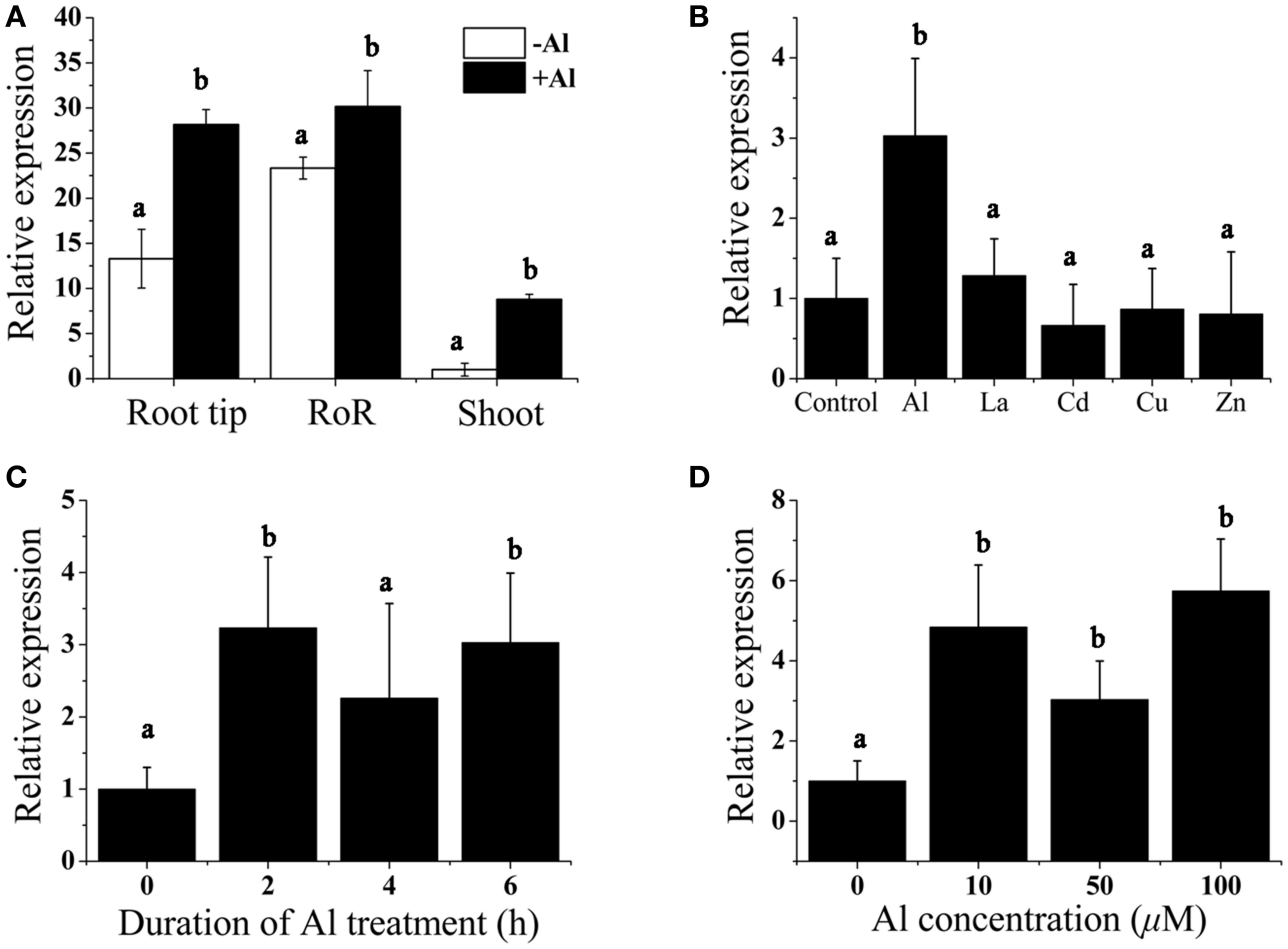
Analysis of *BoALMT1* expression in cabbage seedlings by quantitative real-time PCR. **(A)** Tissue-specific expression of *BoALMT1*. Seedlings were exposed to a 0.5 mM CaCl_2_ solution (pH 4.5) containing 50 μM AlCl_3_ for 6 h. Expression of *BoALMT1* gene in the root tip, rest of root (RoR) and shoots were determined. **(B)** Effect of Al, La, Cd, Cu, and Zn on *BoALMT1* expression. Seedlings were exposed to a 0.5 mM CaCl_2_ solution (pH 4.5) containing 50 μM AlCl_3_, 25 μM Cd, 10 μM La, 0.5 μM Cu, or 2.0 μM Zn. **(C)** Time-dependent expression of *BoALMT1*. Cabbage seedlings were exposed to a solution containing 50 μM AlCl_3_ for different time. **(D)** Dose-response expression analysis of *BoALMT1* gene in cabbage roots. The roots were exposed to a 0.5 mM CaCl_2_ solution (pH 4.5) containing 0, 10, 50, and 100 μM AlCl _3_ for 6 h. Actin expression was used as an internal control. Bars represent means ± *SD* of three replicates and independent experiments were performed at least three times. Different letters above the columns indicate significant differences (*P* < 0.05) between treatments.

We performed real-time reverse transcription (RT)-PCR analysis to measure the expression of *BoALMT1* in the roots and shoots, and found that *BoALMT1* expression was primarily localized to the roots (Figure 2A). Al treatment enhanced its expression in all tissues (Figure 2A). Cabbage plants were exposed to a variety of trivalent cations, and the expression of *BoALMT1* was not induced by lanthanum (La), cadmium (Cd), zinc (Zn), or copper (Cu), but was severely induced by aluminum (Figure 2B). A dose-response experiment and a time-course experiment indicated that increasing the external Al concentration and treatment time did not further increase the *BoALMT1* transcript level (Figures 2C,D).

### Subcellular localization of BoALMT1

**Figure 3:**
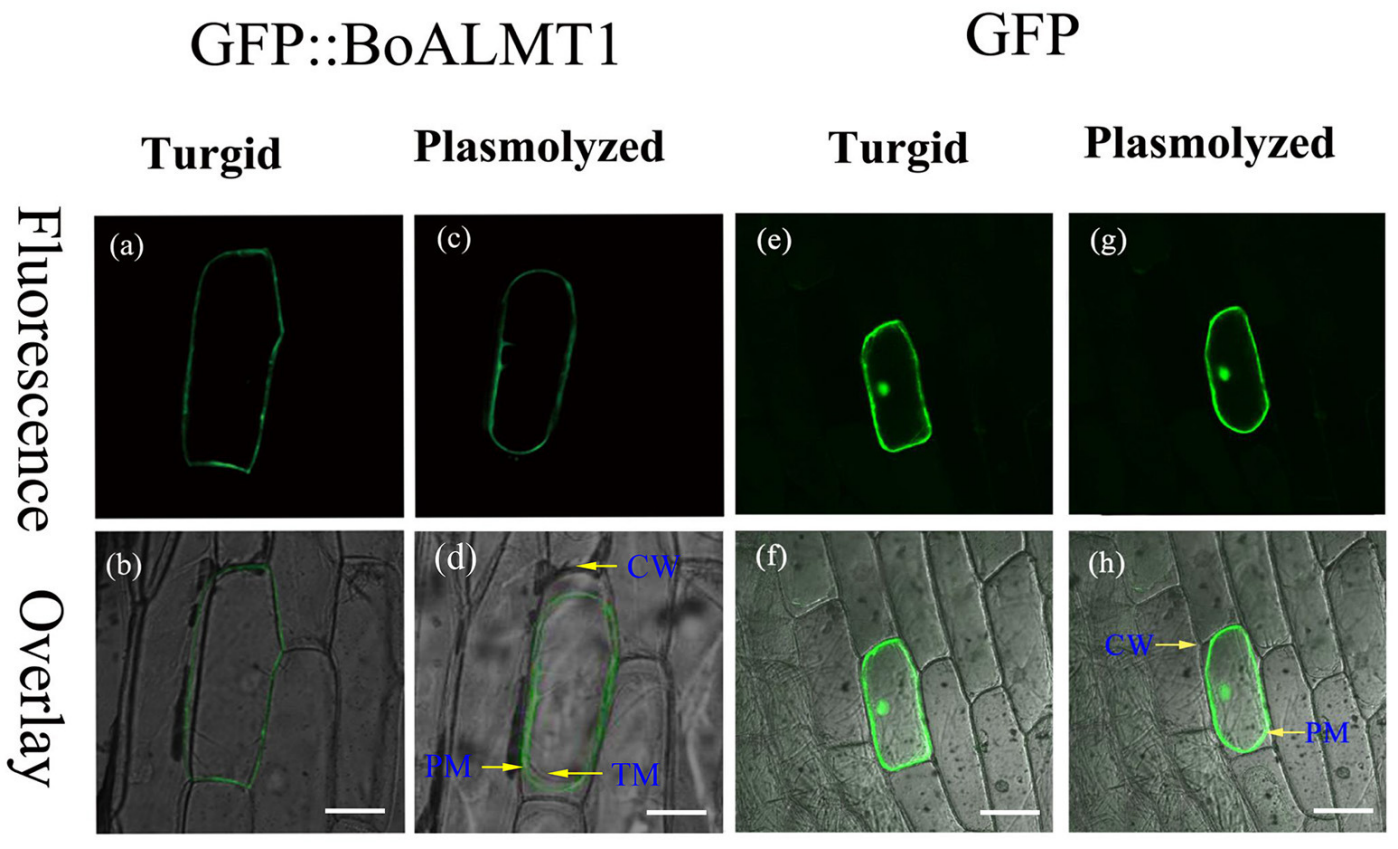
Cellular localization of the BoALMT1 protein by transient expression of the GFP::BoALMT1 fusion protein in epidermal onion cells. **(a–d)** The plasma membrane localization of BoALMT1 in onion epidermal cells before **(a,b)** and after cell plasmolysis with 0.8 M mannitol **(c,d)**. **(e–h)** GFP protein in onion epidermal cells before **(e,f)** and after cell plasmolysis with 0.8M manitol **(g,h)**. PM, CW, and TM labels denote the plasma membrane cell wall and tonoplast membrane localization, respectively. White bars = 100 μm.

The subcellular localization of BoALMT1 was determined via localization of the GFP::BoALMT1 protein transiently expressed in onion epidermal cells (Figure 3). The GFP::BoALMT1 green fluorescence was only observed at the outer layer of the cell (Figures 3a,b), and the cells expressing GFP showed green fluorescence in the whole cell (Figures 3e,f). We induced plasmolysis by the addition of 0.8 M mannitol to distinguish localization in the plasma membrane and observed that the fluorescence of GFP::BoALMT1 was exclusively located in the plasma membrane in the plasmolysis cells (Figures 3c,d). These localization results were similar to those of some ALMTs identified in other species [TaALMT1 (Yamaguchi et al., 2005), BnALMT1 (Ligaba et al., 2006), ZmALMT1 (Piñeros et al., 2008), ZmALMT2 (Ligaba et al., 2008), and GmALMT1 (Liang et al., 2013)].

### Pattern of malate secretion

**Figure 4:**
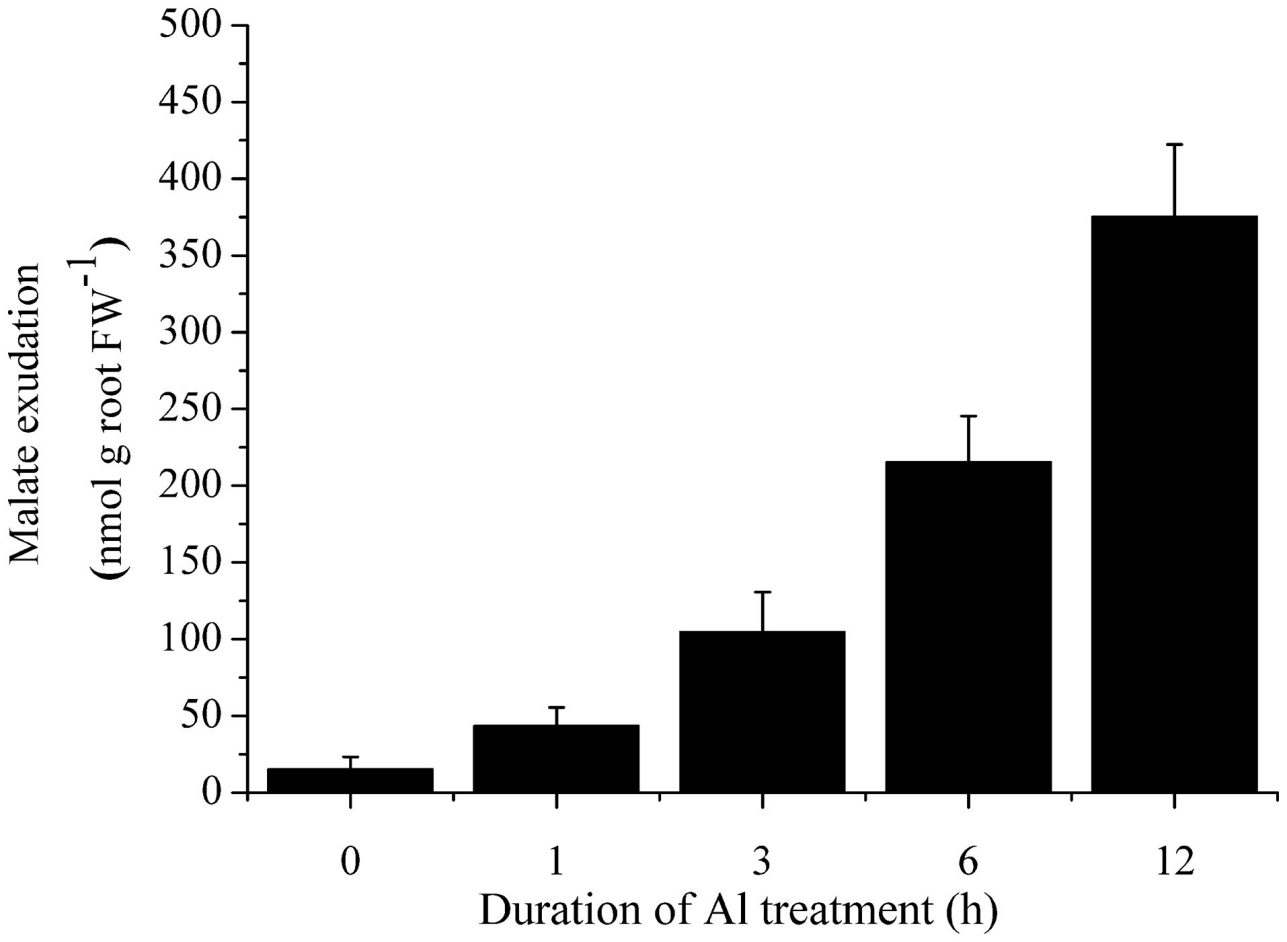
Time course of malate secretion after exposure of 50 μM Al in cabbage roots. Data are means ± *SD* (*n* = 5).

To investigate whether the secretion of malate was induced by Al treatment, we characterized malate exudation from cabbage roots. Cabbage roots secreted a low level of malate under normal conditions. After 3 h treatment with 50 μM Al, malate exudation was remarkably induced (Figure 4).

### Heterologous expression of *BoALMT1* reduced Al-induced H^+^ efflux in *Xenopus* oocytes

**Figure 5:**
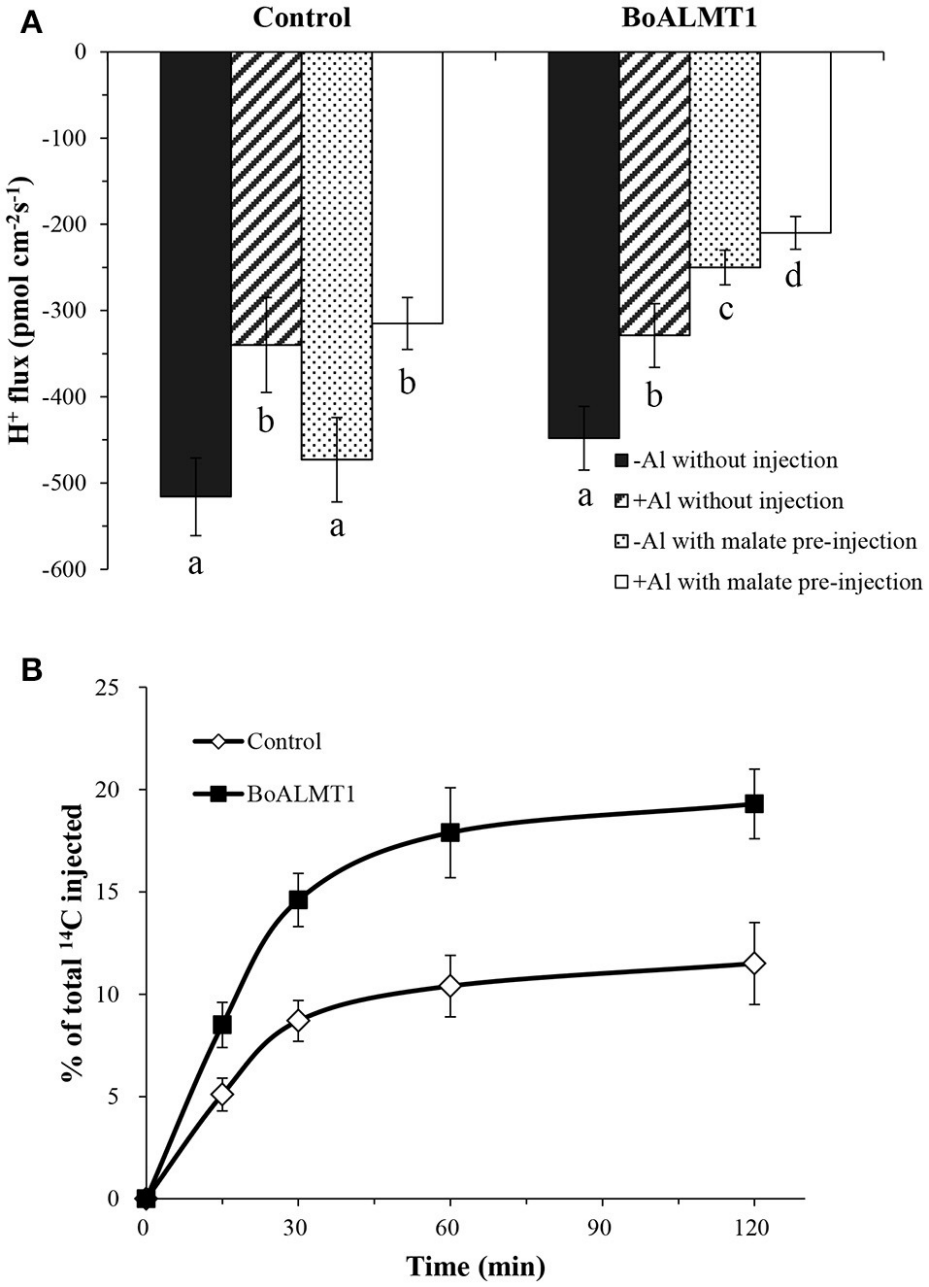
Characterization of BoALMT1 in *Xenopus* oocytes. **(A)** Mean values of H^+^ fluxes in the absence or presence of 50 μM Al with pretreated malate or water. Each value represents the average of at least 3 different cells, the error bars represent *SD* (*n* = 4–6). **(B)** Malate efflux transport activity. Control and *BoALMT1*-expressing oocytes injected with ^14^C-labeled malate were kept in OCM solution. The radioactivity in the bathing solution was measured at the indicated time points; values are expressed as a percentage of the total radioactivity injected. Data was given as means ± *SD* (*n* = 3). Different letters above the columns indicate significant differences (*P* < 0.05) between treatments.

By treated the *Arabidopsis* mutant with Al stress, Degenhardt et al. (1998) observed that the pH of the root surface increased, while Bose et al. (2010) further confirmed Al stress correlated with lower H^+^ influx. So in our study, we used the non-invasive Scanning Ion-selective Electrode Technique (SIET) system to measure H^+^ fluxes crossing the surface of *Xenopus* oocytes with or without the per-injected malate (Figure 5A). We noticed that the H^+^ flux had no difference in the control oocytes under the absence or the present of Al. Furthermore, compared with the control cells, *BoALMT1*-expressing oocytes also secreted similar amount of H^+^ without pretreated with malate. However, when the malate was fed, the *BoALMT1*-expressing oocytes secreted more H^+^ compared with the control oocytes under the absence or the present of Al condition (Figure 5A). To further elucidate BoALMT1 served as a malate efflux transporter, we fed the control and the *BoALMT1*-expressing oocytes with ^14^C-labeled malate and then measured the efflux of radioactively labeled malate (Figure 5B). The *BoALMT1*-expressing oocytes excreted more labeled malate than the control cells. These results indicated that BoALMT1 was a malate efflux transporter and enhanced the H^+^ efflux according to malate secretion in *Xenopus* oocytes.

### Overexpressing *BoALMT1* in *A. thaliana* enhanced Al tolerance

Al-activated membrane transporters, which mediate organic acid release from the root apex, are the primary physiological mechanism of plant Al tolerance (Kochian et al., 2004). Plant ALMTs that have been implicated in malate transport and Al tolerance are TaALMT1 in wheat (Sasaki et al., 2004), AtALMT1 in *Arabidopsis* (Hoekenga et al., 2006), BnAMLT1 and BnALMT2 in oilseed rape (Ligaba et al., 2006), GmALMT1 in soybean (Liang et al., 2013), and MsALMT1 in *M. sativa* (Chen et al., 2013).

**Figure 6:**
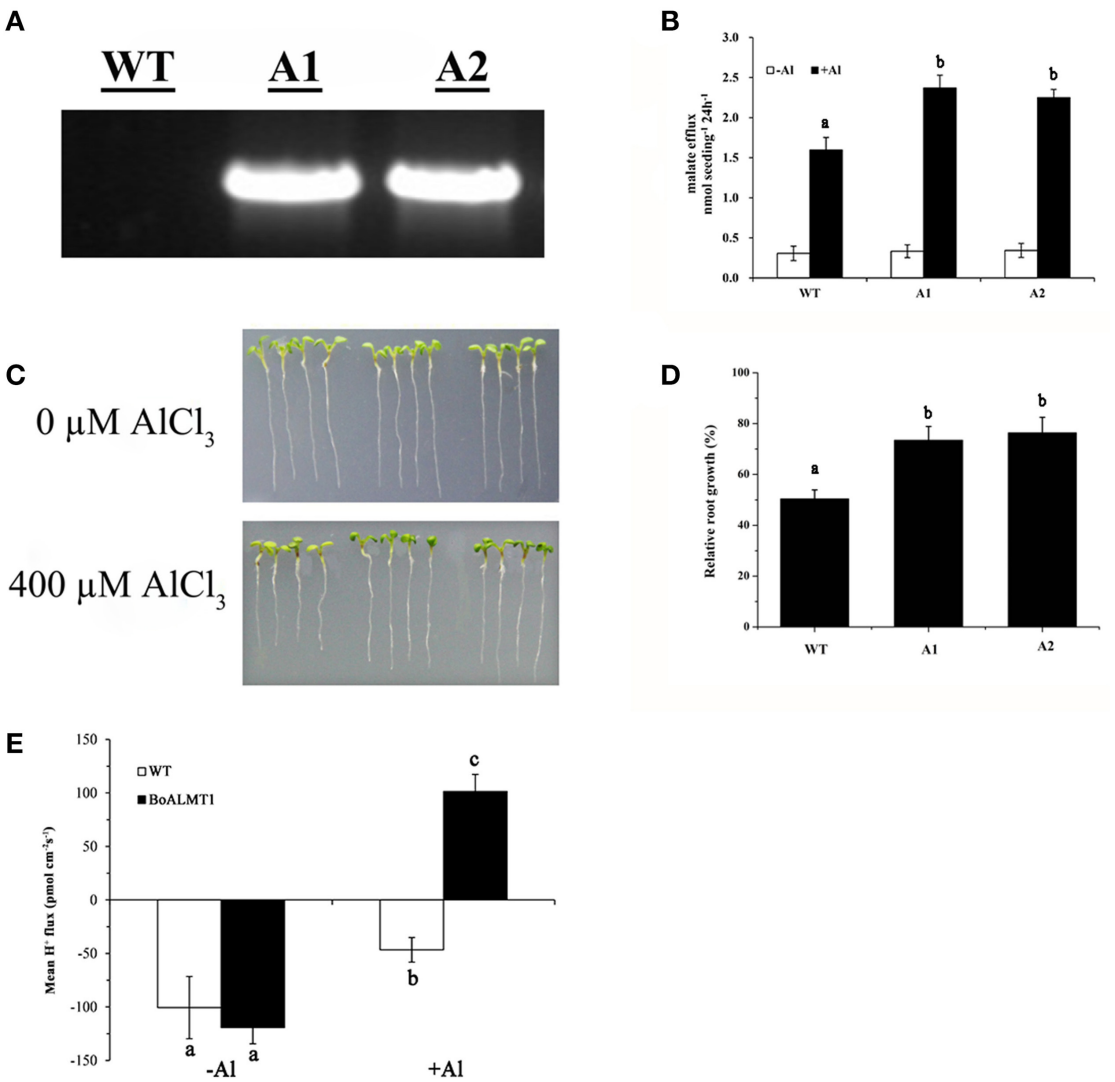
Expression of *BoALMT1* in transgenic *Arabidopsis* plants results in enhanced citrate release and Al tolerance. **(A)** *BoALMT1* expression in the two transgenic lines (A1 and A2) and a control line (WT). **(B)** Root malate exudation in the absence and presence of 50 μM AlCl_3_. Experiments were repeated at least three times (*n* = 100). **(C)** Root growth of representative plants from two independent transgenic lines grown in agar medium in the absence or presence of Al for 2 days. **(D)** Relative root growth of the plants subjected to 400 μM Al for 2 d. Each bar represents the mean of three replicates ± *SD* (*n* = 4). **(E)** Comparison H^+^ flux at the DEZ of 4- to 5-day-old *Arabidopsis thaliana* seedlings in the presence and absence of Al. Data are given as means ± *SD* (*n* = 3–5). Different letters above the columns indicate significant differences (*P* < 0.05) between treatments.

In this study, to investigate whether the overexpression of *BoALMT1* enhances malate exudation and Al tolerance, we induced expression of *BoALMT1* driven by the CaMV 35S promoter in *Arabidopsis* plants. Successful introduction of *BoALMT1* in two transgenic lines, but not the control line, was confirmed by RT-PCR (Figure 6A). Root malate exudation was then measured in the plants expressing *BoALMT1* and demonstrating increased Al tolerance (Figure 6B). Plants expressing *BoALMT1* showed a remarkable increase in root malate exudation rates in the presence of Al, but no difference was observed in the absence of Al. When grown in in agar plates without Al, the transgenic plants expressing *BoALMT1* showed root growth similar to that of wild-type (Figure 6C). When grown in agar plates with 400 μM AlCl_3_, root elongation of plants expressing *BoALMT1* showed less root growth inhibition than that of the plants without expression (Figures 6C,D). To further determine the effect on H^+^ flow caused by overexpressing *BoALMT1* in *Arabidopsis*, we performed SIET to detect the H^+^ flux at the root DEZ with 0 or 50 μM Al (pH = 4.5). Under low pH condition, the pattern of H^+^ influx exhibited no statistic difference between WT lines and *BoALMT1* transgenic lines. However, treated with 50 μM Al, the H^+^ influx was inhibited in the WT lines, while the H^+^ was secreted from the roots *BoALMT1* transgenic lines (Figure 6E).

## Discussion

Al-activated malate transporters (ALMT) have been reported to be involved in Al tolerance and have been isolated from *Arabidopsis, M. sativa*, oil seed rape, rye, wheat, and soybean (Sasaki et al., 2004; Hoekenga et al., 2006; Ligaba et al., 2006; Collins et al., 2008; Liang et al., 2013). Here we reported that Al-induced cabbage BoALMT1 enhanced malate secretion under Al stress in *Arabidopsis*. BoALMT1 contains five predicted transmembrane domains (Figure 1A) and was most closely clustered with BnALMT1 (Figure 1B). The expression of *BoALMT1* was rapidly induced by aluminum and was primarily localized to the root (Figure 2). Some *ALMTs* are Al-induced but not Al-activated, such as *GmALMT1*, but *AtALMT1* is both induced and activated by Al (Hoekenga et al., 2006; Liang et al., 2013). If *BoALMT1* is activated by Al requires further studies.

*BoALMT1* was heterologously expressed in oocytes and *Arabidopsis* to analyze its function (Figures 5, 6). In oocytes, under the absence of Al condition, cells expressing BoALMT1 secreted more H^+^ compared with control cells. After Al treatment, H^+^ influx diminished in the wild type cells and slightly reduced in the *BoALMT1*-expressing cells (Figure 5A). In *Arabidopsis*, the *BoALMT1* overexpression lines exhibited longer root elongation and more malate exudation under Al treatment compared with WT lines, but there no difference between WT and transgenic lines (Figures 6B–D). These results demonstrate that *BoALMT1* increase malate secretion to resist Al tolerance in *Arabidopsis*. This was similar with the reported homologous *ALMT*s in *Arabidopsis* and *B. napus* (Hoekenga et al., 2006; Ligaba et al., 2006). In Figure 6E, compared with the low pH condition, *BoALMT1* expressing plants secreted H^+^ form root tips while the WT plants only diminished the H^+^ influx. As described by Ahn and Matsumoto, the activity of H^+^-ATPase of Al-tolerance wheat lines was higher than that of Al-sensitive wheat under Al treatment (Ahn and Matsumoto, 2006). In faba bean, the activity of PM H^+^-ATPase was increased and positively associated with citrate exudation under Al stress (Chen et al., 2015). The similar results were also found in our previous study about *BoMATE* (Wu et al., 2014). Our results might imply that BoALMT1 mediate malate transport instead of directly mediate H^+^ flux, and the H^+^ efflux might associate with the secretion of malate (Figures 5, 6). However, the causes of these different H^+^ flux patterns between *Xenopus* oocytes and *Arabidopsis* are unclear. Expressing *ALMTs* in yeast and bacteria did not show their functions (Ryan et al., 2011). *BoALMT1* may behave differently in these two heterologous expressing systems. So combining the previous studies by Wu et al. (2014) and Chen et al. (2015) with our findings, we speculated that the secretions of organic acids such as citrate and malate was associated with the activity of PM H^+^-ATPase to resist Al stress.

A C2H2-type zinc finger transcription factor STOP1 plays a key role in plant Al tolerance. Multiple Al-induced genes such as *ALMTs* and *MATEs* are regulated by STOP1 (Liu et al., 2009; Yamaji et al., 2009). To uncover the Al tolerance mechanism in cabbage and determine if STOP1 or a similar regulator participate in this mechanims, further studies are required.

In addition to the external Al detoxification, ALMTs may also have other uncharacterized functions. Recently, Kobayashi et al. demonstrated that *ALMT1* responds to multiple signals such as abscisic acid (ABA), indole-3-acetic acid (IAA), low pH, and hydrogen peroxide, but does not respond to methyl jasmonate and salicylic acid (Kobayashi et al., 2013). A few reports found that aluminum-induced malate efflux is negatively regulated by ethylene by inhibition of the expression of *TaALMT1* (Tian et al., 2014), this process can be alleviated by the inhibition of ACS activity (Yu et al., 2016). Furthermore, TAA1 regulates local auxin biosynthesis and influences the aluminum-induced inhibition of root growth (Yang et al., 2014). Further work should examine the complex regulation of *BoALMT1* during the resistance of multiple stresses and the mechanism by which plants can sense external Al (Kobayashi et al., 2013).

Our results illustrated that the cabbage BoALMT1 localized to the plasma membrane, and the expression of *BoALMT1* was specifically induced by Al treatment. Expression of *BoALMT1* in *Xenopus* oocytes and *Arabidopsis* could enhance Al tolerance. We identified that *BoALMT1* can function as an Al-induced gene, and the BoALMT1 protein is involved in H^+^ flux in response to Al stress.

## Materials and methods

### Plant cultivars and growth conditions

Cabbage (*B. oleracea* cv. Zhonggan-11) was seeded at 25°C on moist filter paper in the dark for 2 days. The seedlings were then moved to a complete nutrient solution (Ligaba et al., 2006). After 5 days of culture, the uniform seedlings were moved to a new plastic pot wetted with 0.5 mM CaCl_2_ (pH 4.5) solution and pre-incubated for ~24 h. To measure the spatial expression patterns of *BoALMT1* in root tips (0–1 cm), after 6 h of 50 μM Al exposure, the roots and shoots were separately collected and subjected to qRT-PCR analysis. To test the specificity of Al-induced *BoALMT1* gene expression, we exposed seedlings in a 0.5 mM CaCl_2_ solution (pH 4.5) containing 50 μM AlCl_3_, 25 μM CdCl_2_, 10 μM LaCl_3_, 0.5 μM CuCl_2_, or 2.0 μM ZnCl_2_ for 6 h. To investigate the dose effects of Al on *BoALMT1* expression, the seedlings were exposed to a 0.5 mM CaCl_2_ solution (pH 4.5) containing 0, 10, 50, or 100 μM AlCl_3_ for 6 h. To analyze time-course effects of Al toxicity on *BoALMT1* expression, the seedlings were exposed to a 0.5 mM CaCl_2_ solution (pH 4.5) containing 50 μM AlCl_3_ for 0, 2, 4, and 6 h.

### Gene cloning and sequencing

To clone *BoALMT1*, RNA was isolated from cabbage seedlings roots treated with Al. To identify cabbage *BoALMT1*, we performed a BLAST search with the known *AtALMT1* and *BnALMT1* sequences on the NCBI website (http://www.ncbi.nlm.nih.gov/). For further amplification, two expressed sequence tags (ESTs) (DK499842 and DY012377) were selected. The two nucleotide sequences were combined to generate a full-length cDNA. The full-length cDNA of *BoALMT1* was amplified with sense primer 5′-ATGGAGAAAGTGAGAGAGATAGTGAG-3′ and anti-sense primer 5′-TCAAATCTGAAGTATACGAACACCC-3′, and then constructed into the pMD18-T vector (Takara, Japan). HMMTOP was used for transmembrane protein prediction analysis. Multiple amino acid alignment was conducted by using ClustalX and MEGA4.1 software.

### Characterization of *BoALMT1* expression via qPCR

*BoALMT1* expression was evaluated using quantitative real-time RT-PCR techniques. Primers for qPCR were designed using Primer 3.0. The first-strand cDNA synthesis was performed by using the Primescript reverse transcriptase (Takara, Japan). We performed real-time PCR with a SYBR Premix Ex Taq™ (perfect real time) kit (Takara, Japan) and using the Applied Biosystems 7500 Real-Time PCR System (ABI) using a relative standard curve method with the following primers: *BoALMT1*, 5′-AGAGAAGGAAGGAGGGTAGGAGAA-3′ (forward) and 5′-GAAGACAACAACGACGGTCA-3′ (reverse); *Actin* (LOC106327159), 5′-TAACAGGGAGAAGATGACTCAGATCA-3′ (forward) and 5′-AAGATCAAGACGAAGGATAGCATGAG-3′ (reverse). Quantitative PCR was performed with conditions of 95°C for 3 min, and then 40 cycles of 95°C for 10 s, 60°C for 30 s, and 72°C for 30 s. Expression data were normalized to the expression level of *Actin* by the ΔΔCt method.

### Subcellular localization of BoALMT1

The subcellular localization of BoALMT1 was determined in onion (*Allium cepa*) epidermal cells. We constructed a vector as 35S:BoALMT1::GFP. The coding region of *BoALMT1* was subcloned into the expression vector pCAMBIA1302 using primers: 5′-CATGCCATGGTAATGGAGAAACTGAGAGAGATAGTG-3′ (forward) and 5′-GGACTAGTAATCTGAAGTATACGAACACCC-3′ (reverse). We transferred the chimera by particle bombardment. The gold particles (1 μm, 1.5 mg) were coated with 5 μg of plasmid DNA in a solution of 2.5 M CaCl_2_ and 0.1 M spermidine (Sigma). We bombarded the epidermal onion peels at a helium pressure of 25–30 Mpa (Bio-rad, U.S.), and then incubated the tissue in MS medium at room temperature in the dark for 24 h. Confocal laser scanning microscopy (Leica DMI 6000B-CS, Germany) with a 488 nm excitation wavelength was used to detect the GFP fluorescence. We induced cell plasmolysis by adding 0.8 M mannitol for 3–5 min.

### *BoALMT1* expression in *Xenopus laevis* oocytes

We cloned the coding regions (cDNA) of *BoALMT1* into the MCS of a pCS107 vector. According to the manufacturer's (Ambion) recommendations, we synthesized the cRNA from 1 μg of *Asc*I-linearized plasmid DNA template. We harvested stage V–VI *Xenopus laevis* oocytes as described previously (Golding, 1992; Hoekenga et al., 2006). We injected 50 nl RNase-free water containing 15 ng of cRNA encoding *BoALMT1* or 50 nl RNase-free water into oocytes using a micro-injector and then incubated the injected oocytes at 18°C for 2 d in oocyte culture medium, OCM; 1L OCM contains 600 ml L-15 (Sigma L4386), 400 mg BSA (Sigma A4919), 5 ml Penicillin-Streptomycin (Gibco 15140-122), and 400 ml H_2_O). Before flux measurements of H^+^, we preloaded malate in the *Xenopus* oocytes by injection of 50 nl of 0.1 M sodium malate or water. Two hours after preloading, the H^+^ fluxes were measured 30 μm away from *X. laevis* oocytes in a solution of 2 mM KCl, 96 mM NaCl, 1 mM MgCl_2_, 0.3 mM MES, 1.8 mM CaCl_2_ with or without 0.1 mM AlCl_3_ and with the pH 4.5. Net H^+^ fluxes were measured using SIET (Xuyue Science and Technology Co., Ltd., Beijing, China) under steady conditions for 8–10 min to insure that no fluctuation was present. We used the OCM bath solution (pH = 4.5) to perform the ^14^C-labeled malate experiment as our previous study (Wu et al., 2014).

### Heterologous expression of *BoALMT1* in *Arabidopsis thaliana*

The coding region (cDNA) of *BoALMT1* was amplified with primers (5′-GCTCTAGAATGGAGAAACTGAGAGAGATAGTG-3′ and 5′-CGCCCCGGGTCAAATCTGAAGTATACGAACACCC-3′) and was cloned into pBI121. We transformed the construct into *Arabidopsis* using *Agrobacterium tumefaciens* via the floral dip method (Clough and Bent, 1998). We used RT-PCR to measure the expression level of *BoALMT1* in the transgenic plants. Root malate release and Al tolerance were analyzed in two independent homozygous transgenic T3 lines as follows. *Arabidopsis* seeds, stratified at 4°C for 3 days, were surface-sterilized and sown onto solid MS medium for 4 days. After germination, we removed uniform seedlings to 0.5 mM CaCl_2_-agar plates containing 0 or 400 μM AlCl_3_ (pH = 4.5). The seedlings were kept on agar plates for 2 days, and then the roots were scanned and the primary root length was measured by the Image J program (Liu et al., 2009). For malate exudation assays, two transgenic *Arabidopsis* and wild-type lines were surface sterilized and germinated on solid MS medium for 1 week. Next, we transferred the seedlings to a 25 ml solution with 0.5 mM CaCl_2_ (pH 4.5) and without Al for 24 h. After this 24 h pre-incubation step, we then transfered the plants to 25 ml exudation medium (pH 4.5) with or without Al (50 μM AlCl_3_). We collected the sample for malate assay by capillary electrophoresis, as described by Hoekenga et al. (2006). We measured the fluxes of H^+^ by using the non-invasive Scanning Ion-selective Electrode Technique (SIET) (Xuyue Science and Technology Co., Ltd., Beijing, China) as described by Bose et al. (2010). The 4- to 5-day-old wild type and BoALMT1 expressing *Arabidopsis* seedlings were equilibrated in a solution (0.1 mM CaCl2, 0.1 mM KCl, 0.3 mM MES, pH 4.5) with or without 50 mM Al for 5–10 min. H^+^ fluxes were measured 200 mm from the root tip for 6–10 min. The H^+^ fluxes were calculated by the JCal V3.1 (a free MS Excel spreadsheet, youngerusa.com or ifluxes.com). The H^+^ flux assay was replicated independently 4–6 times and the data were averaged.

### Statistical analysis

All the statistical analysis was performed by one-way ANOVA and the *t*-test to determine the significance at the *P* < 0.05 level.

## Author contributions

LZ, X-XW, and Y-DG: designed research; X-XW, LZ, JW, CQ, XW, GW, ML, and XL: performed research; JW, X-XW, LZ, and Y-DG: analyzed the data; X-XW, LZ, JW, and Y-DG: wrote the paper.

### Conflict of interest statement

The authors declare that the research was conducted in the absence of any commercial or financial relationships that could be construed as a potential conflict of interest.
